# Indocyanine green fluorescence-guided thoracoscopic pulmonary resection for intralobar pulmonary sequestration: a case report

**DOI:** 10.1186/s13256-019-2128-2

**Published:** 2019-07-27

**Authors:** Nozomu Motono, Shun Iwai, Aika Funasaki, Atsushi Sekimura, Katsuo Usuda, Hidetaka Uramoto

**Affiliations:** Department of Thoracic Surgery, Kanazawa Medical University, 1-1 Daigaku, Uchinada, Ishikawa 920-0293 Japan

**Keywords:** Pulmonary sequestration, Near-infrared, Indocyanine green

## Abstract

**Background:**

The potential complications of pulmonary sequestration are serious and may include recurrent pulmonary infection, hemoptysis, and tumorigenesis. Therefore, the gold standard of treatment has been surgery. Although an adequate boundary between the nonfunctional lung and normal lung is required for the resection of pulmonary sequestration, the boundaries have been conventionally identified intraoperatively with inflation/deflation of the target segment by clamping and unclamping the relevant bronchus. The technique of visualizing the demarcation line based on near-infrared fluorescence imaging with indocyanine green was recently developed.

**Case presentation:**

A 42-year-old Japanese woman with right Pryce III intralobar sequestration was admitted to our hospital. We planned video-assisted thoracoscopic wedge resection of the right sequestration using near-infrared fluorescence imaging with indocyanine green because of the small volume of the nonfunctional region. The aberrant artery was recognized in the pulmonary ligament; the artery was cut off after ligation. Indocyanine green at 5 mg/body was rapidly injected into the peripheral vein, and the boundary of the sequestration was clearly identified under near-infrared fluorescence imaging.

**Conclusion:**

Near-infrared fluorescence imaging with indocyanine green is safe and useful for the identification of the boundary of a pulmonary sequestration.

## Background

Pulmonary sequestration is a rare congenital malformation characterized by nonfunctional lung tissue separated from the normal lung tissue and fed by an aberrant systemic artery [1]. The potential complications of pulmonary sequestration are serious and may include recurrent pulmonary infection, hemoptysis, and tumorigenesis. Therefore, the gold standard of treatment has been surgery. Although thoracotomy has conventionally been required for the resection of pulmonary sequestration, video-assisted thoracic surgery (VATS) has been increasingly frequently performed [2]. Although an adequate boundary between the nonfunctional lung and normal lung is required for the resection of pulmonary sequestration, the boundaries have been conventionally identified intraoperatively with inflation/deflation of the target segment by clamping and unclamping the relevant bronchus. The technique of visualizing the demarcation line based on near-infrared (NIR) fluorescence imaging with indocyanine green (ICG) was recently developed, and its utility was reported [3–13].

We report the safety and utility of ICG fluorescence-guided thoracoscopic pulmonary resection for intralobar pulmonary sequestration.

## Case presentation

**Fig. 1:**
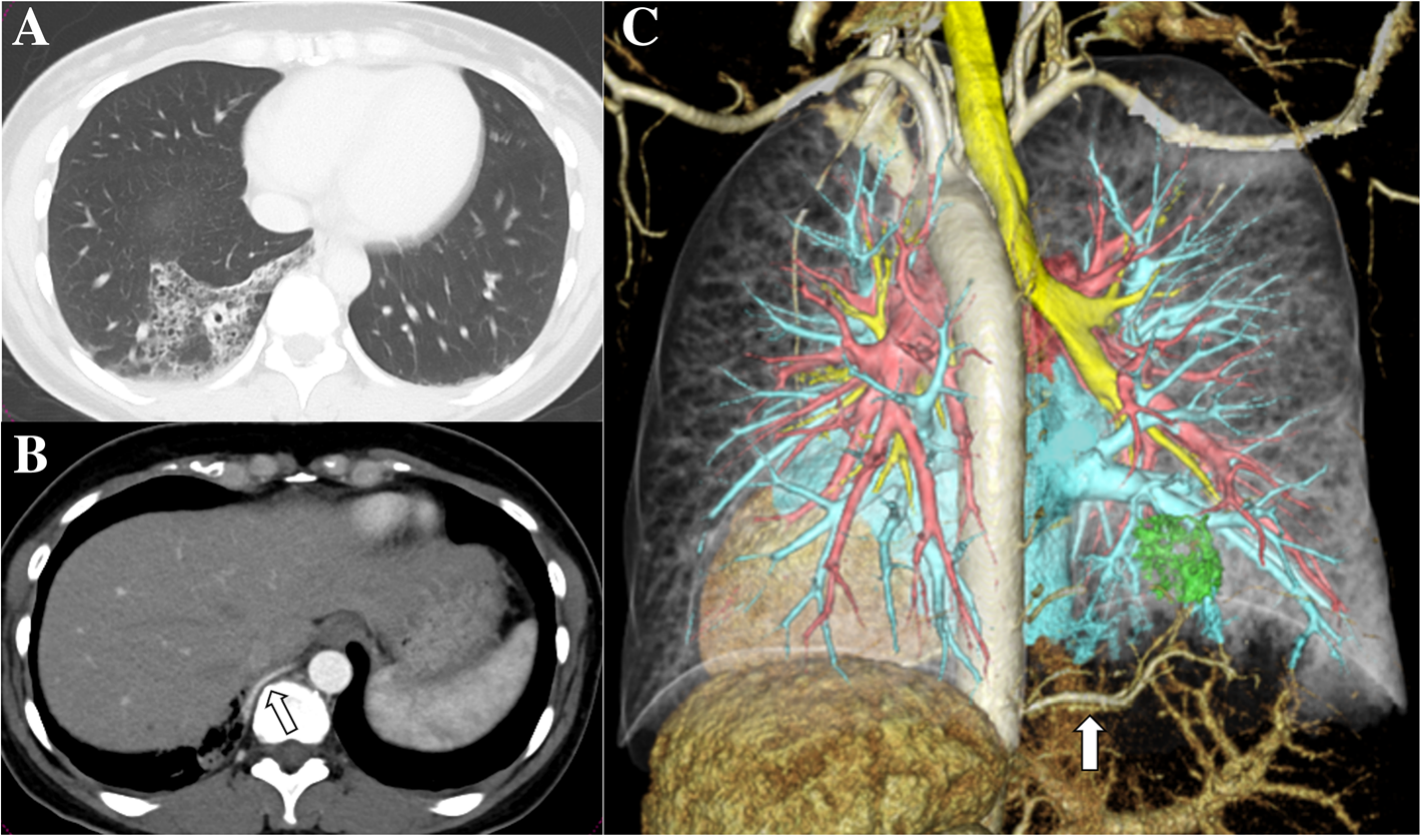
**a** Chest computed tomography showing multicystic changes in the lower lobe of the right lung. **b** Enhanced computed tomography showing the right aberrant artery arising from the descending aorta (*arrow*). **c** Three-dimensional computed tomography showing the right aberrant artery arising from the descending aorta (*arrow*)

An abnormal shadow was detected in a 42-year-old Japanese woman by medical checkup X-ray and she was admitted to our hospital. She had no symptoms and had no medical, social, environmental, obstetrical, family and employment history. She had never smoked tobacco and she occasionally drank alcohol. Her blood pressure was 112/60 mmHg, pulse was 72 beats per minute, and body temperature was 36.4 degrees Celsius. She had no significant abnormal findings on physical and neurological examination. Blood cell count, liver and renal functions, and urine analysis were within normal limits. Any bacteria or fungi were not cultured by microbiological analysis of sputum. Chest computed tomography (CT) showed multicystic change in the lower lobe of her right lung and an aberrant artery arising from the descending aorta (Fig. 1a–c). Because the nonfunctional lung region was covered by normal visceral pleura, she was diagnosed as having Pryce III intralobar sequestration. One and a half months after the first visit, we planned video-assisted thoracoscopic wedge resection of the right sequestration using NIR fluorescence imaging with ICG because of the small volume of the nonfunctional region. The institutional review boards of our hospital approved the protocol (the approval number, M416), and written informed consent was obtained from this patient.

**Fig. 2:**
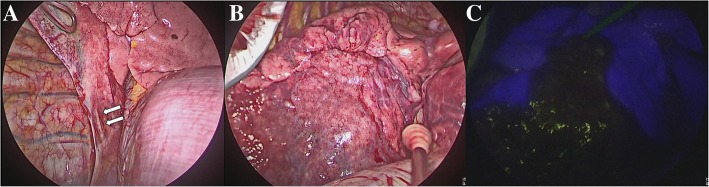
**a** The aberrant artery flows through the pulmonary ligament (*arrows*). **b** The boundary of the sequestration is unclear. **c** The boundary of the sequestration is clearly indicated by indocyanine green

A 3-cm incision was made in the fourth intercostal space, and two additional ports were made in the seventh and ninth intercostal spaces. The aberrant artery was recognized in the pulmonary ligament (Fig. 2a) and cut off using an automatic suture device after ligation. ICG (Daiichi Sankyo Co., Ltd., Tokyo, Japan) at 5 mg/body was rapidly injected into the peripheral vein, and her lung was observed using NIR fluorescence thoracoscopy (IMAGE1 S™; KARL STORZ, Endoscope, Tokyo, Japan). Under the NIR light, the boundary of the sequestration was separated into two areas and then marked on the visceral pleura with electrocautery (Fig. 2b, c). The sequestration lung was stapled based on the boundary line. The operation time was 1 hour and 26 minutes with 5 ml of intraoperative bleeding. Cefazolin sodium at 2 g/day was administered into the peripheral vein for 2 days after surgery, and laboratory findings were within normal limits. Any bacteria or fungi were not cultured by microbiological analysis of lung tissue. No adverse events or complications were seen, and our patient was discharged on postoperative da y 5. This patient has shown no complications in the 1 year since surgery.

## Discussion

This report shows the safety and utility of ICG fluorescence-guided thoracoscopic pulmonary resection for intralobar pulmonary sequestration. Although several studies have reported the utility of identifying the segmental line using NIR fluorescence imaging with ICG [3–13], there are few reports in which ICG was used for identification of demarcation line of intralobar pulmonary sequestration [11]. Management of asymptomatic pulmonary sequestration is controversial. Although surgical resection of pulmonary sequestration has been recommended because of the likelihood of recurrent infection and the possibility of hemorrhage, lobectomy is often required [14]. Although thoracotomy has conventionally been required for resection of pulmonary sequestration, VATS has been increasingly frequently performed [15]. Sublobar resection is usually performed for small sequestration, and it is important to identify an adequate boundary. Traditionally, the boundaries were identified intraoperatively with inflation/deflation of the target segment by clamping and unclamping the relevant bronchus. However, the inflated lung may obstruct the view of the target region, particularly in VATS. The identification rate of the boundary of the target segment ranged from 90–100%. ICG is generally considered safe, and the incidence of severe adverse reactions has been reported to be 0.05% [16, 17]. In previous studies, the dose of ICG used for lung segmentectomy ranged from 0.25 to 5 mg/kg (for example 15 to 300 mg of ICG applied to a patient weighing 60 kg), and no complications were attributed to ICG [3–6, 8, 12]. However, the incidence of anaphylactic shock due to ICG used for angiography at doses of 25 to 75 mg was reported to be 0.05% [17]. Although the dose of ICG was quite low at 5 mg in the present case, the demarcation line of target segment was clearly identified, so this dose of ICG was sufficient for identification of the segmental line. Using such a low dose of ICG is safe, as well, by helping avoid anaphylactic shock.

The difficulty of resection for pulmonary sequestration is the identification of the aberrant artery. Most of the aberrant arteries were present in the inferior pulmonary ligament [18]. Inflammatory changes caused by recurrent infections would develop dense adhesions accompanied with proliferative vessels, and this situation might make the surgical field bloody and blurred. It is important to image the location of the target vessels; three-dimensional CT was useful for identification of the aberrant artery in our case. Because the aberrant artery would be thickened or fragile due to recurrent infections, proximal ligation of the thickened or fragile aberrant artery with a stapling device before cutting it is considered to be important [18].

## Conclusions

NIR fluorescence imaging with ICG was safe and useful for the identification of the boundary for wedge resection of pulmonary sequestration. Low-dose ICG might be sufficient for identification of the segmental line while still avoiding anaphylactic shock. Segmentectomy and wedge resection for small pulmonary sequestration using NIR fluorescence imaging with ICG might become a standard surgical procedure.

## Data Availability

Please contact the corresponding author for data requests.
